# Peer-to-peer accommodation in the time of COVID-19: A segmentation approach from the perspective of tourist safety

**DOI:** 10.1177/13567667221118638

**Published:** 2022-08-15

**Authors:** Marina A. Petruzzi, Catarina Marques

**Affiliations:** Instituto Universitário de Lisboa (ISCTE-IUL), Business Research Unit, Lisbon, Portugal; Unidade de Investigação em Turismo e Hospitalidade, Faculdade de Turismo e Hospitalidade, Universidade Europeia, Lisboa, Portugal; Instituto Universitário de Lisboa (ISCTE-IUL), Business Research Unit, Lisbon, Portugal

**Keywords:** COVID-19, peer-to-peer accommodation, safety measures, tourist segments

## Abstract

This research identifies safety practices to be adopted by organizations of peer-to-peer accommodation for different segments of tourists in a pandemic context. More specifically, it identifies the profiles of tourists based on their opinions on the safety practices they expect to find when booking peer-to-peer accommodation. Results from a Multiple Correspondence Analysis (MCA) and Cluster Analysis applied to a sample of 864 prospective tourists suggest two prominent dimensions of safety practices: information and hygiene, and protection; and four types of tourist segments: concerned tourists, indifferent tourists, forewarned tourists, and confident tourists. While the concerned tourists value all safety practices most, the indifferent tourists do not require access to information about safety measures, although they do want information on the Covid-19 regulations at their destination. The forewarned tourists attach the least importance to aspects such as information and hygiene, and the greatest to the protection aspect. In contrast, the confident tourists value all information practices and safety measures but do not appreciate the protection aspects. These results will be helpful for peer-to-peer accommodation providers wishing to customize services during and after the Covid-19 period.

## Introduction

The sharing economy, specifically peer-to-peer accommodation, has grown enormously in recent years. This has created an alternative model for sharing access to under utilized resources through online platforms, whether for free, for a fee, or for some other form of compensation (Belk, 2014; Frenken & Schor, 2017; Hamari et al., 2016; Wirtz et al., 2019). The sharing economy market was expected to grow from US$15 billion in 2015 to US$335 billion in 2025 (Narasimhan et al., 2018). However, since Covid-19 was declared a public health emergency, the sector entered a period of decline (Zenker & Kock, 2020).

Peer-to-peer accommodation and the whole tourism and hospitality sector are at the heart of the Covid-19 discussion. The industry is considered one of the most affected (Boulos & Geraghty, 2020; Nicola et al., 2020; Tidey, 2020), with small and medium enterprises being exposed to risk (UNWTO, 2020b; Zenker & Kock, 2020). The prolonged lockdown period may also lead organizations into debt, creating an adverse scenario for recovery (Bofinger et al., 2020; Donthu & Gustafsson, 2020). Furthermore, pandemics greatly affect hospitality organizations because they are more vulnerable to health-related crises (McKinsey and Company, 2020).

Covid-19 has affected all aspects of human life (Jones & Comfort, 2020; Verma & Gustafsson, 2020), having reached, as of 30 April 2022, 510,270,667 confirmed cases worldwide, including 6,233,526 deaths (WHO, 2022). From the first cases, many countries and regions started imposing restrictions, such as lockdowns, and the closure of physical stores and businesses to prevent the spread of Covid-19 and to protect healthcare systems (Gössling et al., 2020; Michie, 2020; UNWTO, 2020a).

Many hospitality organizations reviewed their cleaning protocols and implemented new hygiene practices (Bagnera & Stewart, 2020; Zhang et al., 2020). Peer-to-peer accommodation organizations have also attempted to follow this example. In addition to all travel restrictions, the primordial aspect of the peer-to-peer accommodation experience - the host-guest interaction (Acquier et al., 2019; Huber, 2017) – has also suffered from the social distancing imposed by governments (Giritli Nygren & Olofsson, 2020).

The pandemic impacts both the demand and supply sides (Uğur & Akbıyık, 2020; Yang et al., 2020). On the demand side, tourists fear infection, and dealing with new risk perceptions of travel may decrease their willingness to travel (Higgins-Desbiolles, 2020). On the supply side, many tourism and hospitality organizations, such as hotels, restaurants, and museums (Boulos & Geraghty, 2020) have had to close. Tourism, especially in a pandemic context, becomes more sensitive to security and safety issues (Pizam & Mansfeld, 1996), with tourists increasingly worried about the health risks and the spread of the disease. Such aspects may influence tourist behaviour and their decision-making processes (Chinazzi et al., 2020), which results in different impacts on the development and maintenance of tourism and hospitality organizations.

Airbnb is one of the organizations of peer-to-peer accommodation that has been intensely affected by the pandemic, with a 96% drop in bookings (DuBois, 2020) and a decrease in its value from $31 billion in 2017 to $18 billion by April 2020 (Evans, 2020). The organization has struggled with refunds and guest cancelations (Evans, 2020). It is predicted that Airbnb will recover, although it was expected that the organization would have an issue with supply because some hosts will have moved back to the long-term rental market to avoid risk (Dolnicar & Zare, 2020). Further, some experts contend tourists may favour traditional hotels over peer-to-peer accommodation due to hygiene standards and social distancing, even after the pandemic has been brought under control (Hossain, 2020). Industry analysts consider hotels will have an advantage over peer-to-peer accommodation in a post-Covid period due to the lack of standardization (Glusac, 2020). The fact that such a situation could arise supports the relevance of a study that helps peer-to-peer accommodation hosts develop practices to address the lack of regulation regarding safety measures. This current research aims to understand tourists’ opinions on this subject and to segment them in such a way as to make it easier for hosts to deal with any issues and improve safety standards.

Previous research in the era of Covid-19 with regard to peer-to-peer accommodation has focused chiefly on how hosts perceive the impact of the pandemic (Farmaki et al., 2020a), how this affects their decision-making process (Zhang et al., 2021), and tourist consumption during Covid-19 (Jang et al., 2021). Less attention, however, has been paid to tourists’ opinions. And no research has segmented tourists according to their opinions on the safety measures they expect to find during an overnight stay at peer-to-peer accommodation. Thus, by understanding the new scenario that has emerged from the Covid-19 pandemic and the challenges organizations face, this study aims to identify the practices organizations of peer-to-peer accommodation have to adopt in order to address tourists’ expectations in terms of safety measures based on different segments of tourists. More specifically, it examines what measures make different categories of tourists feel secure in their choice of peer-to-peer accommodation when travelling during and after a pandemic period.

It is crucial to investigate tourism's basic unit (tourist) to identify which segments to target and what strategies may benefit the sector's future. As not everyone has had identical attitudes and behaviours towards the pandemic's evolution, peer-to-peer organizations must implement these practices according to groups of tourists with similar characteristics. Therefore, based on a theoretical framework built on travel constraints and safety measures and two propositions, a tourist segmentation process was undertaken in two analytical phases with 864 prospective tourists. In the first phase, a Multiple Correspondence Analysis (MCA) was applied to understand the practices that best discriminate the tourists. In the second phase, a Cluster Analysis, more precisely the K-means cluster method, was conducted to segment the tourists. This is an important undertaking, given that to our knowledge, tourist clustering has not been developed in the accommodation sector, particularly in peer-to-peer accommodation in a pandemic context. This business model involves direct interaction between residents and tourists who mutually share spaces such as bathrooms and kitchens; it requires specific and possibly different safety measures than those adopted in other types of accommodation, such as hotels. This will be useful for peer-to-peer accommodation providers with regard to customizing services during and after the Covid-19 period.

## Literature review

### Peer-to-peer accommodation and safety aspects

Peer-to-peer accommodation is defined as the space available in private homes for tourist accommodation, offered by a non-commercial host to a user for a short period of time, and mediated by online platforms (Dolnicar, 2019a; Wirtz et al., 2019). In the interaction, there is direct contact between host and guest (Dolnicar, 2019a), who may develop relationships and meaningful social encounters (Belk, 2010; Molz, 2013).

Among the various organizations of peer-to-peer accommodation, Airbnb appears as the most well-known organization. It offers individuals the opportunity to share spare space as accommodation for travellers (Guttentag, 2019). The organization states its benefits to all stakeholders, suggesting that individuals can have a genuine, inclusive, and sustainable experience (Airbnb, n.d.). Airbnb offers accommodations in more than 220 countries and regions worldwide, with over seven million listings (Airbnb, 2020a). More recently, as the newest organization, Fairbnb.coop emerged on the marketplace. It intends to work with municipal authorities and invest back in local projects (Fairbnb.coop, n.d.). One of the main goals and motivations behind the organization is the sustainability of the host communities, particularly in terms of the social aspects (Petruzzi et al., 2021). Fairbnb.coop presents itself as a non-extractive alternative to peer-to-peer accommodations, prioritizing people over profit. The organization offers hosts and guests an authentic, sustainable, and meaningful experience, allowing the development of social projects within host communities (Fairbnb.coop, n.d.).

Like many other hospitality and tourism organizations, peer-to-peer accommodation has also implemented a wide range of measures to mitigate the impact of Covid-19 (Hossain, 2020). It provides tourists with experiences that incorporate safety principles, which is a paramount objective of tourism organizations and destinations (Reisinger & Mavondo, 2006). New insurance policies and safety measures, more thorough cleaning processes to be followed by hosts, and orientations in terms of disinfectants to be used for cleaning the properties became necessary implementations. Additionally, organizations implemented a 24-h gap between guests’ bookings (Hossain, 2020; Wood, 2020), provided guidance with information on Covid-19 prevention (Wood, 2020), and suggested that hosts avoid physical contact with guests by not being present at check-in whenever possible (Airbnb, 2020b).

Peer-to-peer accommodation organizations have also implemented reimbursement policies to support guests in cases of cancelations (Shuk, 2020; Webster, 2020). Fairbnb.coop, for instance, offers refundable bookings in case of cancelation; the social project, which receives half of the commission, still receives its crowdfunding share (Fairbnb.coop, 2020). Airbnb offers a variety of cancellation and refund options depending on a set of eligible criteria (Shuk, 2020).

Overall, safety aspects have become an essential part of the operation in peer-to-peer organizations, which should make tourists feel more comfortable with peer-to-peer accommodation than hotel stays (Mckinsey and Company, 2020). Hosts will probably need to provide a cleaning demonstration or follow a list of protocols to convince guests of their hygiene and safety measures (Hossain, 2020). In addition, organizations will have to provide insurance for cancelations as this has become one of the aspects travellers consider when planning a trip (Uğur & Akbıyık, 2020).

### Travel constraints and safety system theory

Some streams of research address safety, risk, and constraints. Many tourism scholars (e.g. Nyaupane & Andereck, 2008; Nyaupane et al., 2004) have investigated travel constraints by incorporating leisure constraint concepts. This is linked to factors that inhibit tourist travelling or negatively impact the travel experience (Hung & Petrick, 2010). Previous literature suggests three types of travel constraints: intrapersonal constraints, interpersonal constraints, and structural constraints (Crawford et al., 1991; Wilson & Chen, 2020). Travel constraints can influence tourists’ behaviour and decision-making and they will probably look for measures to minimize the constraints faced (Huang & Hsu, 2009; Hung & Petrick, 2010).

Other tourism scholars identify the patterns of safety management from the perspective of a system, adopting the safety system theory (Leveson 2011; Lower et al., 2018). Previous literature suggests that the safety system elements involve: human safety capabilities; the safety and reliability of equipment and environments; safety functions of the energy production process; and safety information flow (Xie et al., 2021).

The proliferation of Covid-19 has increased travel constraints and raised safety issues, mainly because of travel bans and restrictions (Pan et al., 2021). More specifically, the pandemic has increased intrapersonal constraints, with one of the sub-constructs being individuals’ safety during travel (Hung & Petrick, 2010). Therefore, given the proliferation of Covid-19, it is urgently necessary to understand the safety element from the tourist perspective, as this will allow the definition and implementation of measures that make tourists feel safe in peer-to-peer accommodation when they travel.

### Tourist perceived safety

Safety, as a basic need for tourists, has been highlighted by much of the literature on hospitality and tourism. Safety is a representative issue in tourists’ decision-making (Seabra et al., 2013); it is built around the perception of risk at the destination, even if not directly related to tourism (Pappas & Glyptou, 2021). Consequently, strengthening safety measures is critical to the sustainable development of many organizations in the tourism and hospitality context. Travel safety concerns the degree of risk a tourist can tolerate during travel (Xie et al., 2021); in other words, travel that does not involve threat, loss, or injury (George, 2010). The assessment of such conditions constitutes what Chauhan (2007) calls "tourist perceived safety at destinations," which is measured by asking tourists to express their opinions regarding a sense of safety on issues such as staying in accommodations (George, 2003).

Safety opinions can be understood as feelings of safety and protection from risks. Xie et al. (2021) classify tourist perceived safety under four dimensions: (i) perceived safety of human elements; (ii) perceived safety of facility and equipment elements; (iii) perceived safety of environmental elements; and (iv) perceived safety of management elements. Irrespective of dimension, the personal safety of tourists represents a significant impact on tourism demand (Seabra et al., 2013). Therefore, safety can be seen as a fundamental condition for developing destinations (Fletcher & Morakabati, 2008). Strategies to make tourists feel safe before and during travel are essential for organizations to remain in business and for the success of destinations (Huan & Beaman, 2004).

In recent times, the Covid-19 pandemic has given tourists worldwide more reason for concern about safety issues. Tourism, due to its cross-border nature, introduces new diseases that can spread around the world, and the very risk of disease can change tourists’ behaviour (Rittichainuwat & Chakraborty, 2012). This is mainly because many fear risk to their health through the possibility of contracting disease (Chinazzi et al., 2020). A recent study suggests that the Covid-19 pandemic can change tourists’ travel habits and affect how they think and feel (Zenker & Kock, 2020). Studies have analysed the opinion of tourists on aspects of safety and risk (Marine-Roig & Huertas, 2020; Xie et al., 2020). In an environment where risk is compounded by a pandemic, it is more than ever important to understand tourists’ opinions as they represent, within the tourism and hospitality literature, a subjective interpretation which reveals how their judgments and attitudes towards products, destinations, and organizations are made (Swarbrooke & Horner, 1999). Furthermore, research has shown that tourists will not spend money in places where they do not feel safe (Pizam & Mansfeld, 1996). Their consumer behaviour is influenced by sanitation and hygiene (Kaushal & Srivastava, 2021).

Having reviewed the literature, we set out in the following section a theoretical framework and the propositions to be tested.

### Theoretical framework and propositions

Research highlights that tourists focus on personal safety and seek to feel safe and secure when travelling (Feickert et al., 2006). Safety measures refer to customers’ protection from potential injury or death (Enz, 2009), and the literature considers that most of them fall into three categories: hygiene, physical safety, and management (Xie et al., 2021).

**Figure 1.**
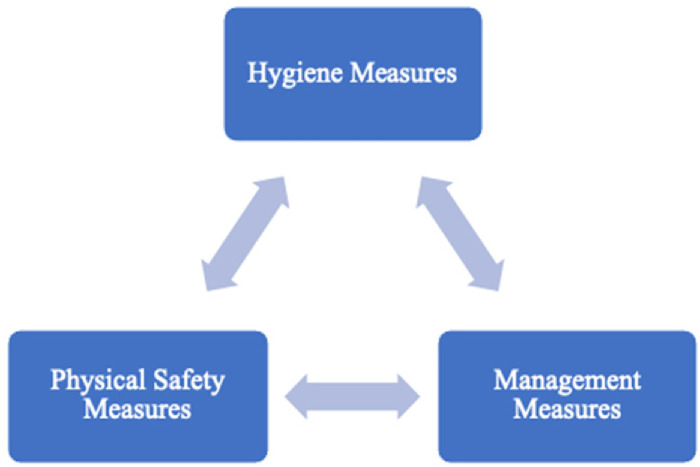
Pandemic safety measures framework for peer-to-peer accommodation.

Focusing on a pandemic context, a theoretical framework (Figure 1) is proposed to show and analyse the main safety measures for peer-to-peer accommodation. The framework integrates the three fundamental categories of safety measures that emerged from the literature, and that are considered most relevant for the purposes of this study. Xie et al. (2021) classify tourist perceived safety under four dimensions, which we have adapted and applied in this study. The dimension “perceived safety of social environment”, for instance, encompasses aspects of the “hygiene measures” category; the “perceived safety of facility and equipment element” encompasses aspects of the “physical safety measures” category; and the “perceived safety of management elements” encompasses aspects of the “management measures” category.

#### Hygiene measures

Research on hygiene measures or perceived safety of social environment (as classified by Xie et al. (2021)) refers to deep cleaning and hygiene protocols (Aiello et al., 2020; Farmaki et al., 2020b; Pavlatos et al., 2020; Samanci et al., 2020). It has informed scholars seeking to better understand ways to ensure tourists’ safety (Meng et al., 2021). Many tourism and hospitality-related businesses must focus mainly on hygiene and cleanliness to reassure travellers that they are safe (Meng et al., 2021) during an experience, such as peer-to-peer accommodation.

This is of paramount importance, particularly but not exclusively, in a pandemic context. The absence of such hygiene measures can compromise the tourist experience, but when implemented successfully, they can improve the perception of safety (Volgger et al., 2021).

#### Physical safety measures

Research on physical safety measures or perceived safety of facility and equipment elements (as classified by Xie et al. (2021)) refers to the safety assessment of protective equipment, social distancing, and remote check-in and check-out (Bagnera & Stewart, 2020; Fong et al., 2020; Hossain, 2020; Ketter & Avraham, 2021; Uğur & Akbıyık, 2020). They represent the set of facilities and equipment that may enhance the safety of tourists during an overnight stay at peer-to-peer accommodation.

Implementing these measures is considered crucial to boosting control and trust during Covid-19 (Volgger et al., 2021), mainly because many tourists are concerned about the safety standards of particular equipment and facilities in the accommodation (Xie et al., 2021).

#### Management measures

Research on management measures or perceived safety of management (as classified by Xie et al. (2021)), which encompasses safety management policies and aspects related to managerial levels, has indicated such measures as elements essential to supporting regular tourism activity. Organizations should implement management initiatives such as the issuing of certificates, behavioural safety elements (e.g. information about Covid-19), and safety system elements (e.g. financial guarantees in case of cancelation) (Bagnera & Stewart, 2020; Hossain, 2020; Shin & Kang, 2020; Uğur & Akbıyık, 2020).

The evidence uncovered by examining the literature on tourist safety measures supports the development of the following proposition:

**Proposition 1:** Measures that make tourists feel safe in peer-to-peer accommodation can be grouped into hygiene, management, and physical safety measures.

Tourists have different opinions, behaviour, motivations, and needs (Tussyadiah & Pesonen, 2017). Such heterogeneity calls for the identification of safety measures that differentiate the distinct segments of tourists. At the same time, it is essential to examine tourists’ opinions on the safety measures in order to find practices that minimize the extent of damage caused by the Covid-19 pandemic. This study, therefore, not only identifies these measures but also groups the tourists in the context of peer-to-peer accommodation. Thus, a second proposition is formulated:

**Proposition 2:** Different tourists perceive safety measures in peer-to-peer accommodation in different ways, i.e., there are different groups of tourists regarding safety practices.

### Tourist segmentation

Segmentation consists in dividing the market into smaller and more homogeneous segments that present similar needs, motivations, and/or characteristics (Dolnicar, 2019b). Such segments may demonstrate similar behaviours, which is helpful with regard to adapting marketing policies (Weinstein, 1994). The criteria applied for segmentation may be based on physical or behavioural attributes and on a single consumer characteristic or a set of them (Dolnicar et al., 2018). The purpose of the analysis and the market under study will guide the segmentation type to be adopted (Wedel & Kamakura, 2000).

Market segmentation is used as a tool in the decision-making process to select the target market for a specific product or service (Tynan & Drayton, 1987). Successful organizations drive their business based on segmentation (Lilien & Rangaswamy, 2004). The main advantage of targeting segments is that an organization can position itself to offer the particular products or services that suit a specific segment, which can help it develop a long-term competitive edge. A further advantage is that communicating with a reduced group of tourists may reduce marketing expenses. Segmentation offers a better understanding of the differences between consumers (Dolnicar, 2019b).

Previous studies have sought to segment tourists who use peer-to-peer accommodation. In one, Tussyadiah and Pesonen (2017) identified two profiles of tourists based on their motivations for using peer-to-peer accommodation in Finland and the United States. In a motivation-based segmentation, Guttentag et al. (2018) identified five segments of tourists who chose Airbnb.

With regard to safety measures, Rittichainuwat (2013) segmented tourists into four groups based on their perceptions of safety measures, type of accommodation, and purpose of visit.

## Methodology

### Sample and data collection

The study's target population consists of prospective tourists of peer-to-peer accommodation organizations. Data came from a comprehensive survey on respondents’ opinions about potential actions to be implemented in rented places both during the Covid-19 period and regular times. The survey was conducted by Fairbnb.coop. Participants were invited to participate via the organization's social platform (https://fairbnb.coop/get-involved/) and other online social media platforms such as Facebook and LinkedIn. An online self-administered questionnaire consisting mainly of multiple-choice questions was developed in English and applied by the organization to three types of stakeholders (prospective tourists/guests, hosts, and social project proponents). Questions regarding the Covid-19 period included aspects such as safety measures, holiday choices in the following six months, main motivations to travel, the choice between urban or rural areas, and a hotel or apartment stay, among others. Questions regarding travel during regular times related to measures to be implemented for service improvement. The survey also covered travel habits, sustainability preferences when choosing accommodation, and demographics like age and country of residence. Some survey variables were not used in the data analysis as they were not within the scope of the current study.

Data were gathered between July and September 2020, and a non-probabilistic sample of 1016 participants was obtained. From this sample 864 (85%) were assumed to be prospective tourists. Although the data were collected by Fairbnb.Coop, the items selected for analysis are not specifically related to this organization but refer to safety aspects of peer-to-peer accommodation in general, during a pandemic period.

### Variable selection regarding safety aspects

Guidance for selecting items to be analysed emerged from the literature on peer-to-peer accommodation and safety aspects in a pandemic. Included were items relating to measures of hygiene (e.g. deep cleaning, list of hygienic measures, hygiene protocols) (Aiello et al., 2020; Farmaki et al., 2020a), management (e.g. cleaning certification, information about Covid-19 regulations, financial insurance against cancellations) (Shin & Kang, 2020; Uğur & Akbıyık, 2020) and physical safety (e.g. online check-in/check-out, protective equipment) (Fong et al., 2020; Hossain, 2020).

Overall, 11 items pertained to the three aspects presented in the theoretical framework. These items were assessed by the organization team and the researchers to confirm their appropriateness regarding the constructs’ face validity i.e., the degree to which the 11 items subjectively appear to measure the safety constructs. The items were grouped in the questionnaire using two multiple-choice questions. The items were defined as nominal for the analysis, with the categories: (a) chosen and (b) not chosen. Table 1 presents the list of items.

**Table 1.** Frequencies for the opinions on safety practices (Total and per cluster).

		Cluster								Total		Pearson Chi-Square
		1 Concerned Tourists		2 Indifferent Tourists		3 Forewarned Tourists		4 Confident Tourists				
		N	%	N	%	N	%	N	%	N	%	
Deep cleaning before each check-in	*Chosen*	173	90.6%	232	74.8%	75	79.8%	227	84.4%	707	81.8%	21.462*
	*Not Chosen*	18	9.4%	78	25.2%	19	20.2%	42	15.6%	157	18.2%	
Masks, gloves and sanitation gel	*Chosen*	158	82.7%	35	11.3%	74	78.7%	48	17.8%	315	36.5%	373.949*
	*Not Chosen*	33	17.3%	275	88.7%	20	21.3%	221	82.2%	549	63.5%	
Mandatory gap day(s) between bookings	*Chosen*	109	57.1%	93	30.0%	25	26.6%	161	59.9%	388	44.9%	76.283*
	*Not Chosen*	82	42.9%	217	70.0%	69	73.4%	108	40.1%	476	55.1%	
Remote/Automated check-in and check-out	*Chosen*	88	46.1%	36	11.6%	19	20.2%	94	34.9%	237	27.4%	82.400*
	*Not Chosen*	103	53.9%	274	88.4%	75	79.8%	175	65.1%	627	72.6%	
Having the place sanitized and verified by a professional cleaning company	*Chosen*	106	55.5%	16	5.2%	38	40.4%	22	8.2%	182	21.1%	231.399*
	*Not Chosen*	85	44.5%	294	94.8%	56	59.6%	247	91.8%	682	78.9%	
List of hygiene measures to follow at the apartment	*Chosen*	166	86.9%	67	21.6%	16	17.0%	199	74.0%	448	51.9%	306.002*
	*Not Chosen*	25	13.1%	243	78.4%	78	83.0%	70	26.0%	416	48.1%	
Mandatory hygiene protocols for Hosts	*Chosen*	164	85.9%	76	24.5%	19	20.2%	230	85.5%	489	56.6%	338.628*
	*Not Chosen*	27	14.1%	234	75.5%	75	79.8%	39	14.5%	375	43.4%	
Allow a direct contact with the host before booking	*Chosen*	117	61.3%	144	46.5%	43	45.7%	148	55.0%	452	52.3%	12.808*
	*Not Chosen*	74	38.7%	166	53.5%	51	54.3%	121	45.0%	412	47.7%	
Insurance that covers damage from cancellations	*Chosen*	172	90.1%	137	44.2%	89	94.7%	77	28.6%	475	55.0%	244.834*
	*Not Chosen*	19	9.9%	173	55.8%	5	5.3%	192	71.4%	389	45.0%	
Information about Covid-19 regulations of the visited country	*Chosen*	178	93.2%	172	55.5%	55	58.5%	244	90.7%	649	75.1%	146.160*
	*Not Chosen*	13	6.8%	138	44.5%	39	41.5%	25	9.3%	215	24.9%	
Highlighting Hosts that are committed to meet all standards of hygiene	*Chosen*	155	81.2%	55	17.7%	28	29.8%	183	68.0%	421	48.7%	253.117*
	*Not Chosen*	36	18.8%	255	82.3%	66	70.2%	86	32.0%	443	51.3%	

*p < 0.01.

### Data analysis procedure

This study investigates the inter-relationships among the safety variables through an exploratory data analysis, applying a MCA (Gifi, 1996). The MCA allowed us to describe the associations between the multiple variables about the tourists’ opinions and, consequently, to identify the variables that best discriminate tourists’ opinions. It also provided a map of all the categories along two axes, the MCA dimensions, and graphically displayed the associations amongst variables.

A Cluster Analysis was subsequently performed to classify tourists according to their personal opinions. A typology of the different opinions was created using the MCA dimensions as input variables and the K-Means Clustering method. Clusters obtained were named according to the tourists’ opinions about safety measures and profiled in terms of holiday choices and travel motivations (sets of multiple-choice questions transformed into nominal variables with binary categories - chosen and not chosen) during the pandemic period and tourists’ characterization as well. As most variables are nominal or ordinal (e.g. age), qui-square tests of independence were conducted to identify differences among clusters.

## Results

### Sample characterization

Tables 1, 2, 3, and 4 present the frequency distribution of the tourist's opinions and characterization. The sample consists of 864 respondents assumed to be prospective tourists of peer-to-peer accommodation organizations. Of these, 65% are between the ages of 30 and 60, and around 20% are older than 60 (see the total columns in Table 3). Regarding their travel habits, tourists travel with their partner (47%), their family (21%), alone (17%), or with friends (15%). Most of them consider finding sustainable solutions when travelling, to minimize the negative effects (75%) (see the total columns in Table 3). Respondents are mainly people living in the USA (18%), UK (13%), Italy (11%), France (9%), Spain (9%), Canada (7%), Germany (6%), among others (see the total column in Table 4).

**Table 2.** Frequencies for the tourists’ holidays choices and travel motivation variables during the pandemic period (Total and per cluster).

		Cluster								Total		Pearson Chi-Square
		1 Concerned Tourists		2 Indifferent Tourists		3 Forewarned Tourists		4 Confident Tourists				
		N	%	N	%	N	%	N	%	N	%	
**In the next 6 months you are probably going to**												
Stay home and have day trips or short trips in your surroundings.	*Chosen*	130	68.1%	154	49.7%	42	44.7%	175	65.1%	501	58.0%	29.094 *
	*Not Chosen*	61	31.9%	156	50.3%	52	55.3%	94	34.9%	363	42.0%	
Have Holidays close to home.	*Chosen*	89	46.6%	97	31.3%	28	29.8%	100	37.2%	314	36.3%	
	*Not Chosen*	102	53.4%	213	68.7%	66	70.2%	169	62.8%	550	63.7%	
Have Holidays in other parts of your country.	*Chosen*	94	49.2%	154	49.7%	51	54.3%	133	49.4%	432	50.0%	
	*Not Chosen*	97	50.8%	156	50.3%	43	45.7%	136	50.6%	432	50.0%	
Have Holidays also abroad.	*Chosen*	41	21.5%	90	29.0%	28	29.8%	61	22.7%	220	25.5%	
	*Not Chosen*	150	78.5%	220	71.0%	66	70.2%	208	77.3%	644	74.5%	
**Main Motivations to travel**												
Visiting family and friends	*Chosen*	116	60.7%	156	50.3%	50	53.2%	139	51.7%	461	53.4%	13.528 *
	*Not Chosen*	75	39.3%	154	49.7%	44	46.8%	130	48.3%	403	46.6%	
Business trips	*Chosen*	31	16.2%	40	12.9%	11	11.7%	31	11.5%	113	13.1%	
	*Not Chosen*	160	83.8%	270	87.1%	83	88.3%	238	88.5%	751	86.9%	
Attending Festivals / Events	*Chosen*	34	17.8%	41	13.2%	7	7.4%	40	14.9%	122	14.1%	
	*Not Chosen*	157	82.2%	269	86.8%	87	92.6%	229	85.1%	742	85.9%	
Discovering new places	*Chosen*	155	81.2%	236	76.1%	84	89.4%	219	81.4%	694	80.3%	
	*Not Chosen*	36	18.8%	74	23.9%	10	10.6%	50	18.6%	170	19.7%	
Learning more about my culture	*Chosen*	48	25.1%	64	20.6%	29	30.9%	65	24.2%	206	23.8%	
	*Not Chosen*	143	74.9%	246	79.4%	65	69.1%	204	75.8%	658	76.2%	
Enjoying nature	*Chosen*	145	75.9%	210	67.7%	65	69.1%	214	79.6%	634	73.4%	
	*Not Chosen*	46	24.1%	100	32.3%	29	30.9%	55	20.4%	230	26.6%	
**Where do you feel more comfortable staying?**	*Urban*	59	31.1%	91	29.7%	32	34.4%	73	27.2%	255	29.8%	3.749
	*Suburban*	11	5.8%	22	7.2%	9	9.7%	22	8.2%	64	7.5%	
	*Rural*	120	63.2%	193	63.1%	52	55.9%	173	64.6%	538	62.8%	
**Do you feel more comfortable staying in a hotel or in an Apartment / Holiday Home?**	*Hotel*	39	20.7%	35	11.4%	15	16.3%	32	11.9%	121	14.1%	10.136**
	*Apartment/Holiday Home*	149	79.3%	273	88.6%	77	83.7%	236	88.1%	735	85.9%	

*p < 0.01; **p < 0.05.

**Table 3.** Frequencies for the tourists’ characterization variables (Total and per cluster).

		Cluster								Total		Pearson Chi-Square
		1 Concerned Tourists		2 Indifferent Tourists		3 Forewarned Tourists		4 Confident Tourists				
		N	%	N	%	N	%	N	%	N	%	
**What can you tell us about your travel habits?**	*travel alone*	25	13.8%	48	16.3%	16	17.0%	53	20.6%	142	17.2%	9.431
	*travel with the family*	31	17.1%	70	23.8%	18	19.1%	55	21.4%	174	21.1%	
	*travel with the friends*	27	14.9%	47	16.0%	14	14.9%	33	12.8%	121	14.6%	
	*travel with partner*	98	54.1%	129	43.9%	46	48.9%	116	45.1%	389	47.1%	
**And how old are you?**	*under 20*	0	0.0%	1	0.3%	0	0.0%	3	1.1%	4	0.5%	40.744 *
	*21–30*	32	16.8%	50	16.2%	21	22.3%	31	11.5%	134	15.6%	
	*31–40*	37	19.5%	83	26.9%	34	36.2%	55	20.4%	209	24.3%	
	*41–50*	28	14.7%	62	20.1%	15	16.0%	47	17.5%	152	17.7%	
	*51–60*	45	23.7%	71	23.1%	13	13.8%	71	26.4%	200	23.2%	
	*older*	48	25.3%	41	13.3%	11	11.7%	62	23.0%	162	18.8%	
**Which of the following statements describes you best?**												
*When I travel I want to enjoy myself and forget the responsibilities of my daily life.*		11	5.9%	10	3.3%	10	10.8%	8	3.0%	39	4.6%	28.062*
*I love travelling. but I am concerned about the negative effects. That's why I try to find sustainable solutions if possible*		123	66.5%	234	78.3%	68	73.1%	202	76.2%	627	74.5%	
*I focus on the best offer. I am happy if it is sustainable but if not I would still take it*		35	18.9%	29	9.7%	9	9.7%	23	8.7%	96	11.4%	
*I love travelling but not at any cost, if there are no sustainable solutions - I am out!*		16	8.6%	26	8.7%	6	6.5%	32	12.1%	80	9.5%	

*p < 0.01.

**Table 4.** Distribution of respondents by Country (Total and per Cluster) (for more than 1.5% of respondents).

Total			Cluster 1 Concerned Tourists			Cluster 2 Indifferent Tourists			Cluster 3 Forewarned Tourists			Cluster 4 Confident Tourists		
Country	N	%	Country	N	%	Country	N	%	Country	N	%	Country	N	%
USA	155	18.0	USA	46	24.5	Italy	46	15.1	Italy	21	22.3	USA	56	20.8
UK	109	12.7	UK	27	14.4	USA	43	14.1	Spain	16	17.0	UK	39	14.5
Italy	95	11.1	Spain	18	9.6	UK	35	11.5	USA	10	10.6	Canada	31	11.5
France	77	9.0	France	16	8.5	France	34	11.1	UK	7	7.4	France	21	7.8
Spain	73	8.5	Canada	15	8.0	Germany	23	7.5	France	6	6.4	Spain	16	5.9
Canada	61	7.1	Italy	13	6.9	Spain	23	7.5	Germany	6	6.4	Germany	15	5.6
Germany	51	5.9	Australia	10	5.3	Belgium	15	4.9	Canada	4	4.3	Italy	13	4.8
Belgium	32	3.7	Germany	7	3.7	Swiss	13	4.3	Belgium	3	3.2	Swiss	12	4.5
Swiss	31	3.6	Belgium	6	3.2	Canada	11	3.6	Swiss	3	3.2	Australia	11	4.1
Australia	27	3.1	Netherlands	5	2.7	Netherlands	11	3.6	Greece	2	2.1	Netherlands	9	3.3
Netherlands	26	3.0	Swiss	3	1.6	Portugal	9	3.0	Ireland	2	2.1	Belgium	8	3.0
Portugal	14	1.6				Australia	6	2.0	Poland	2	2.1	Mexico	4	1.5
Ireland	12	1.5				Ireland	5	1.6				Sweden	4	1.5
						Mexico	5	1.6						

When asked about travelling during the pandemic, most respondents prefer to stay at home and have day or short trips (59%) or have holidays in other parts of the home country (50%). Only a quarter of the respondents stated that they want to have holidays abroad. Discovering new places (80%), enjoying nature (73%), and visiting family and friends (53%) are the primary motivations for travelling. More than half of them feel more comfortable staying in an apartment or holiday home (85%), and rural areas (63%) instead of urban areas (30%) (see the total columns in Table 2).

Deep cleaning before each check-in is the most chosen safety measure (82% of respondents chose it), followed by Information about the Covid-19 regulations at the destination country. Having the place sanitized and verified by a professional cleaning company and remote/automated check-in and check-out were considered the least important. These measures were chosen by only 21% and 27% of the respondents, respectively (see the total columns in Table 1).

### Typologies of tourists

An MCA was carried out to identify the dimensions that best discriminate the tourists’ profiles based on their opinions on safety practices during the Covid-19 period. An initial set of 11 input variables was used (see Table 1). Two dimensions were identified, the discrimination measures and contributions of which are presented in Table 5. Two variables were removed from the analysis due to low discrimination values (“Allow direct contact with the host before booking”, “Deep cleaning before each check-in”). Although “Mandatory gap day(s) between bookings” and “Remote/Automated check-in and check-out” have low discrimination measures, they were considered to be part of dimension 1 as their discrimination measures in this dimension are slightly lower than the dimension inertia.

**Table 5.** Discrimination and relative contribution of variables (a).

	Dimension			
	1	%	2	%
Masks, gloves and sanitation gel	**0.225**	10.3	**0.233**	20.7
Mandatory gap day(s) between bookings	0.158	7.2	0.024	2.1
Remote/Automated check-in and check-out	0.201	9.2	0.000	0.0
Having the place sanitized and verified by a professional cleaning company	0.160	7.3	**0.200**	17.8
List of hygiene measures to follow at the apartment	**0.346**	15.9	0.075	6.7
Mandatory hygiene protocols for Hosts	**0.378**	17.3	0.104	9.2
Insurance that covers damage from cancellations.	0.064	2.9	**0.445**	39.5
Information about Covid-19 regulations of the visited country.	**0.259**	11.9	0.029	2.6
Highlighting Hosts that are committed to meeting all standards of hygiene.	**0.390**	17.9	0.016	1.4
Total (eigenvalue)	2.181	100.0	1.126	100.0
Inertia	0.242		0.125	

(a) Values of discrimination measures of each dimension higher than the dimension inertia are highlighted in bold.

Based on the variables with the highest discrimination measures, it can be observed that while the characteristics related to information on hygiene measures and regulations are decisive in the first dimension, having cancellation insurance, protective equipment, or a sanitization guarantee by a professional cleaning company stand out in the second dimension. These latter two variables are simultaneously important in both dimensions. In summary, while the first dimension is defined by items that appeal to the importance of information and hygiene, the second dimension has protection as a reference.

**Figure 2.**
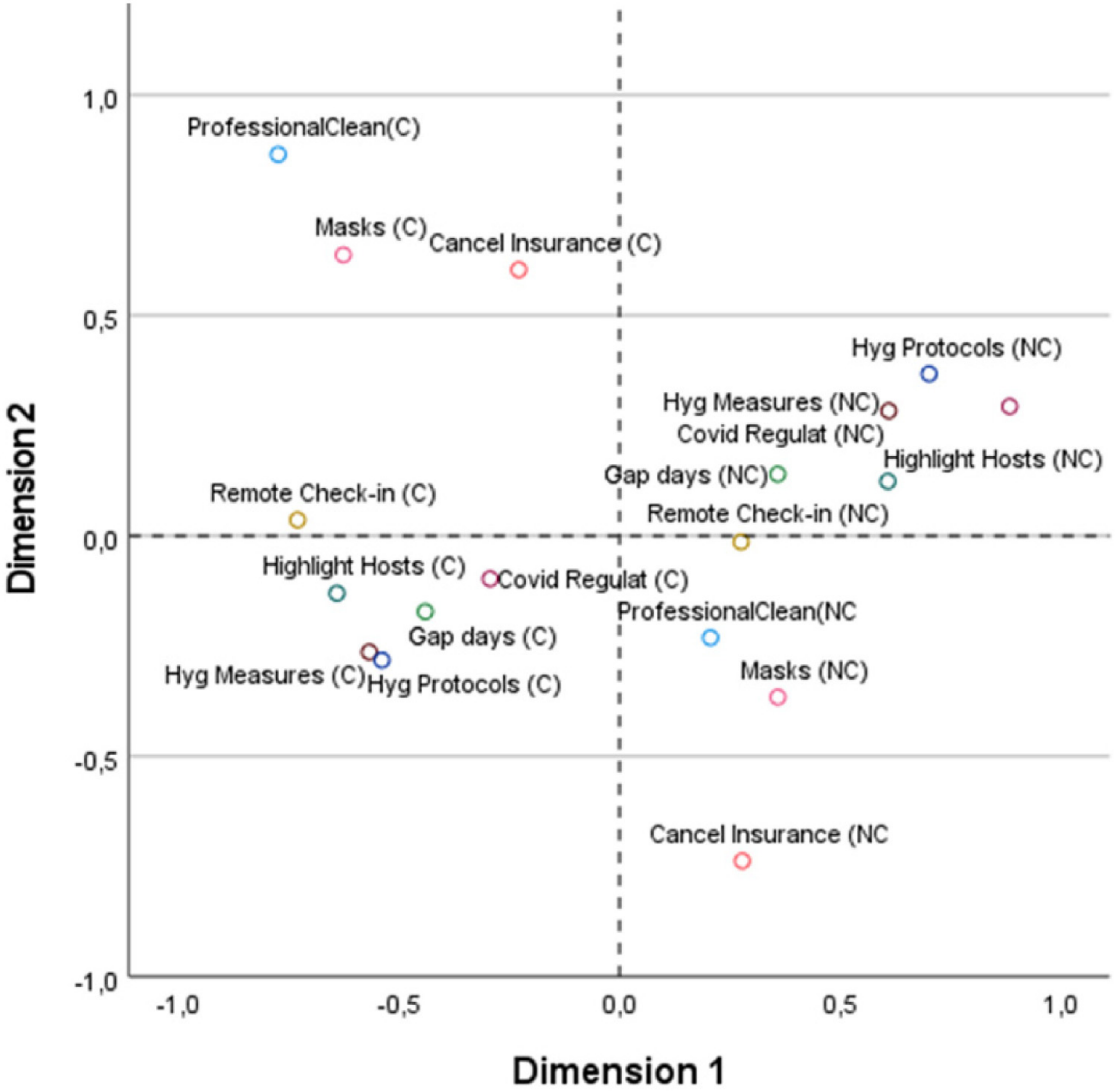
Topological configuration of tourists’ profiles. (a) C = Chosen, NC = Not Chosen.

Figure 2 shows the associations among categories (chosen vs. not chosen) of the multiple variables displayed in the two dimensions. The results show that there are patterns of associations between categories that can induce the presence of tourists who tend to share the same opinion. Thus, different groups of associations will correspond to groups of tourists with different profiles. Dimension 1 contrasts respondents who chose and those who did not choose factors related to information and hygiene. In turn, dimension 2 differentiates tourists regarding the protection aspects of sanitization and insurance, contrasting those who consider these aspects important with those who do not. Therefore, the results suggest four typologies of tourist profiles.

A cluster analysis was carried out to classify tourists according to their personal opinions. The K-means clustering method was used to estimate a solution of four clusters based on the object scores resulting from the MCA safety practices dimensions. The tourist profile of each cluster was based on the mean of the object scores (see Table 6) and on the distribution of the tourists’ opinions and characterization (see Tables 2, 3, and 4).

**Table 6.** Mean of dimension's object scores for each cluster.

	Cluster			
Dimension	1 N = 191; 22.1%	2 N = 310; 35.9%	3 N = 94; 10.9%	4 N = 269; 31.1%
1- Information and Hygiene	−1.215	0.967	0.323	−0.365
2- Protection	0.666	−0.004	1.566	−1.016

Cluster 1 (22% of respondents) was named “Concerned Tourists” because it comprises tourists who attach the most significant importance to all the aspects related to dimension 1, such as information on hygiene measures and Covid-19 regulations at the destination and hosts having mandatory hygiene protocols. They also consider important the existence of individual protective equipment, the existence of insurance against cancellation and the cleaning being done or verified by a specialized company. They are the ones who most chose the option of remote check-in and check-out (46%). Regarding holidays during the pandemic period, more than 2/3 of these respondents intend to stay home and have day or short trips in their home surroundings, and almost half want to have holidays close to home (47%). This group comprises the oldest tourists, as 49% of its members are over 50 years old. Most of them are from the USA (25%), UK (14%), Spain (10%) and France (9%). Regarding the choice of sustainable solutions when booking, this tourist group's profile can be considered the least sustainability friendly of the four profiles because with regard to finding sustainable solutions only 67% chose this option, and 19% chose the best offer instead.

Cluster 2 is the largest group, composed of 36% of respondents. It was named “Indifferent Tourists” as they do not consider access to information about the measures important. However, they want to know information about the Covid-19 regulations in the destination country (just over 50%). They attach no importance to the existence of individual protective elements, having cancellation insurance, and having the cleaning carried out by professional companies. When asked about holidays, half stated that they intend to stay home, but 30% will probably holiday close to home. Most of these tourists are between 30 and 60 years old (70%). They are mainly from Italy (15%), the USA (14%), the UK (12%) and France (11%).

Cluster 3 is the smallest group (11% of respondents). Its members attach less importance to information and hygiene aspects (dimension 1); they just want to be aware of the Covid-19 regulations at the destination (59%). However, they attach the most significant importance to the protection aspects, given that 95% chose the existence of cancellation insurance and 80% attach importance to the presence of individual protective equipment (masks, gloves, and sanitation gel) at the accommodation. Therefore, this cluster was named “Forewarned Tourists”. These tourists are those who more chose to have holidays in other parts of their own country (54%), although 45% wanted to stay at home to have day or short trips in the home surroundings. They are those who more stated that they would not like to have holidays close to home (70%) and who more want to travel abroad (30%). These are the youngest tourists, with 60% between 21 and 40 years old. Most of them are from Italy (22%), Spain (17%) and the USA (11%).

Cluster 4 (31% of respondents) is the opposite of cluster 3. These potential tourists attach importance to all information practices and hygiene measures, but contrastingly, they are the ones who least value the aspects of individual protection, insurance against cancellation, and specialized cleaning. In addition, 60% of the members of this group believe that there should be gaps between bookings. For these reasons, this cluster was called "Confident Tourists." Regarding holidays, they stated that they want to stay home (65%) or have holidays close to home (37%). Like cluster 1, it is composed of the oldest tourists, given that about half of the group are over 50 years old, and are tourists from the USA (21%) and UK (15%). There is also a small but relevant percentage of members from Canada (12%) and France (8%). Tourists in this cluster can be considered sustainability friendly, as 75% of them are concerned about the negative effects of travel and try to find sustainable solutions; in addition, 12% of these tourists do not travel if this kind of solution does not exist

There is no difference among the four segments of tourists regarding travel motivations. Discovering places and enjoying nature are the main motivations, and they all feel more comfortable in rural areas. Clusters 1 (concerned tourists) and 3 (forewarned tourists) have some members who said they prefer to stay in a hotel rather than an apartment during this period (21% and 16%, respectively).

## Conclusion and implications

This study identifies measures to make tourists feel safe and choose peer-to-peer accommodation when travelling during and after a pandemic period. In accordance with the literature on travel constraints and safety issues, a theoretical framework is proposed, and two propositions are defined. Overall, the findings indicate two main dimensions – information and hygiene, and protection; and four separate tourist segments – concerned tourists, indifferent tourists, forewarned tourists, and confident tourists. Hosts and peer-to-peer organizations can use the profiles of these different segments to determine which practices to adopt to address tourists’ demands and therefore survive during the pandemic and post-pandemic period.

Contrary to what was formulated in proposition 1, prospective customers associate the safety measures with two dimensions. Hygiene and management measures are not differentiated but aggregated in dimension 1, highlighting the need for information. Customers want to be aware of the hygiene measures applied at the accommodation and the Covid-19 regulations at the destination. They also want there to be certification for hosts committed to high standards of hygiene. Other safety measures related to hygiene concerns are identified in this first dimension. In turn, dimension 2 encompasses items relating to protective measures present in the groups of physical safety and management measures.

**Figure 3.**
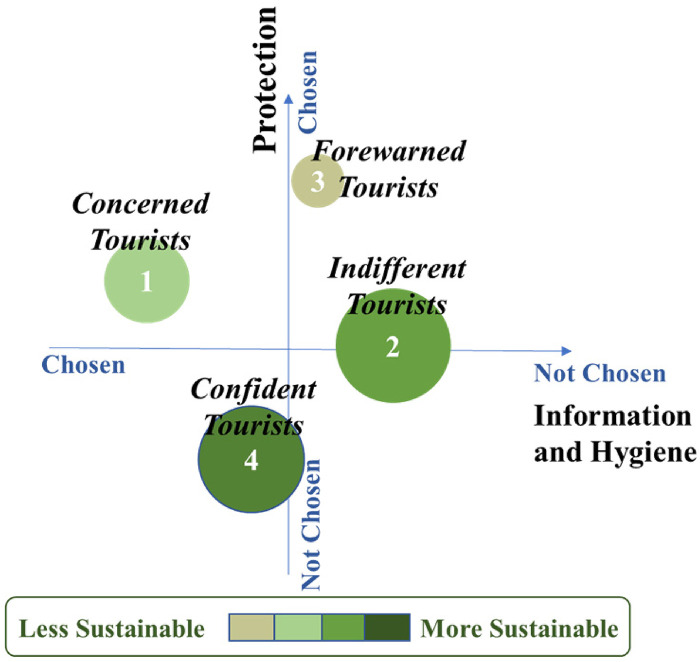
Topological representation of Clusters, Sustainable preferences and Size. Size of each bubble is proportional to the cluster size.

Figure 3 presents the four clusters topologically for their better characterization in terms of safety measures, sustainability preferences, and importance/size to peer-to-peer accommodation organizations. Understanding tourists’ sustainability preferences is crucial, especially because sustainability is a topic that appears repeatedly in the COVID-19 literature (Yang et al., 2021).

With regard to safety practices, all four clusters of tourists are identified, verifying proposition 2. The concerned tourists attach the greatest importance to information and hygiene protocols, local Covid-19 regulations, and the services of a specialized cleaning company, along with the availability of protective equipment and insurance against booking cancellations. The latest measures are reinforced by Uğur and Akbıyık (2020), who analysed general travellers’ comments in a pandemic context, and by Canina and McQuiddy-Davis (2020), who studied guests’ appreciation of mask-wearing. Remote check-in and check-out are also measures much welcomed by this profile of tourists, reinforcing that the avoidance of physical contact between hosts and guests should be adopted by hospitality organizations (WTTC, 2020).

In contrast, the indifferent tourists do not care about information on the hygiene measures adopted by hosts, although they do appreciate having access to information related to local regulations on Covid-19. They also do not attach importance to the availability of protective equipment, travel insurance, or the services of a professional company certifying cleanliness. This segment of tourists, who represent the biggest cluster, does not require peer-to-peer accommodation organizations to adjust other practices besides the information about Covid-19 regulations of the country.

In turn, the forewarned tourists most appreciate the availability of protective equipment, such as masks, gloves, and sanitation gel, and insurance against cancellations. This finding is reinforced by Statista’s (2020) survey, which pointed to travellers’ willingness to even pay more for the possibility of having cancellation flexibility. Despite both aspects of protection, this segment of tourists attaches less importance to information aspects, being only concerned with knowing the Covid-19 regulations in the visited country.

Conversely, the *confident tourists* want to know all the information about practices and hygiene, although they are less concerned about individual protective equipment, insurance against cancellations, and the services of a specialized cleaning company. They also appreciate gaps between guests’ overnight stays, as reinforced by Hossain (2020). To meet the expectations of this tourist segment, organizations of peer-to-peer accommodation should work with hosts to provide a wide range of information about the practices and measures adopted at the accommodation.

In contrast to Seabra et al. (2013), the cluster analysis allows us to identify tourists from different countries who may have similar opinions about security measures. The reason for this may be because the analysis is conducted with regard to the accommodation and not the destination. Nevertheless, the analysis is made in the context of the Covid-19 pandemic, which is worldwide. Therefore, it may represent a similar impact factor even in different destinations and contexts.

It is recommended that when implementing practices, peer-to-peer accommodation organizations should not only take into account the opinions of different target markets, but also define which practice is most relevant for each group of tourists. It is common sense that the practice of deep cleaning should be adopted for all the four groups of tourists and in every type of accommodation, while protective equipment, such as masks, for example, should be available in those accommodations shared with hosts and/or other guests.

Identifying measures that make the tourist feel safe in peer-to-peer accommodation can provide advantages for organizations and hosts when preparing strategies for the sector impacted by pandemics like Covid-19 (Qiu et al., 2020). In addition, health and safety influence accommodation bookings, not only for the elderly but also for the whole population (Pappas & Glyptou, 2021).

### Theoretical implications

Although hospitality organizations have been previously investigated in a pandemic context (e.g. Dolnicar & Zare, 2020; Farmaki et al., 2020a), this study makes valuable contributions to the literature on peer-to-peer accommodation during pandemics such as Covid-19. First, it offers an innovative analysis of tourists’ profiles, which resulted in two main dimensions of importance: (1) information and hygiene and (2) protection; and four tourist segments - (i) concerned tourists; (ii) indifferent tourists; (iii) forewarned tourists; and (iv) confident tourists. Second, to the best of our knowledge, this study is the first attempt to identify tourist segments based on safety practices in a pandemic context. As such, it advances the theoretical understanding of safety measures to be adopted based on tourists’ opinions, and segments them accordingly. Third, it deepens knowledge on travel constraints and safety issues, which are key areas for tourists’ decision-making and the sustainable development of destinations.

### Practical implications

As practical implications, the profiles of tourist segments can be helpful to peer-to-peer accommodation organizations and hosts. The obtained profiles guide the practices to be adopted to meet tourists’ expectations on safety measures when booking an accommodation. As such, when working on their strategies, practitioners should adopt practices that meet the requirements of their target guests. The consequence of not doing so could lead to a reduction in the number of bookings and a paucity of guests, intensifying the negative impact of Covid-19 on peer-to-peer accommodation. The contribution this study makes, in light of the sharing economy and, more specifically, peer-to-peer accommodation, having been so hard hit by Covid-19 (Brammer et al., 2020), is that it informs organizations of the practices to adopt in order to attract guests and ensure they feel safe about overnighting in this type of accommodation, during and after a pandemic period. Consequently, it reinforces Konak (2022), who considers it is important that tourism organizations implement hygiene and safety measures to increase tourists’ perception of security.

To meet the expectations of concerned tourists, practitioners should provide information for guests and ensure that hosts adhere to the required health and safety standards. Organizations must invest in current technologies to inform tourists during the booking process and also at the accommodation, as suggested for hotels (Shin & Kang, 2020; Stergiou & Farmaki, 2021; Yu et al., 2021). For instance, pop-ups could be used on the platform to keep guests informed about the Covid-19 regulations in the country and region being booked. This information should also be included in the booking confirmation sent by email. Another appropriate strategy to provide guests with information about the cleaning, hygiene measures and protocols adopted in the apartment would be to display a QR (Quick Response) code at the property. This way, tourists can check updated data on their phones. Importantly, this strategy reflects past guidance from Hacker et al. (2020) and the WHO (2020), who suggested that hotels should ensure distance safety, health and hygiene protocols when designing customer experiences.

As a complementary measure, it is highly recommended that organizations work on and reinforce their insurance against cancellations at the point of booking to provide explicit guarantees for their guests. Organizations may offer hosts and guests individual protective kits of masks, gloves, and sanitation gel to improve safety. At the same time, specialized cleaning companies accredited by the organization could be hired by hosts. This reinforces suggested strategies for hotels with regard to providing a management model to ensure customer safety and hygiene (Gössling et al., 2020; Robina-Ramírez et al., 2021).

As the *indifferent tourists* care only about the information on local regulations, the suggested QR-Code at the apartment and making information available on the platform during the booking process may be enough to make these tourists feel safe to book peer-to-peer accommodation. In turn, for the *forewarned tourists*, practitioners should focus primarily on insurance policies against cancellations and provide individual protective kits. These practices tend to make tourists feel safe about booking peer-to-peer accommodation. To meet the expectations of the *confident tourists,* who attach importance to information and gap days between bookings, organizations may implement the aforementioned QR-Code and a mandatory gap of at least 24 h, for disinfection and ventilation, between guests’ overnight stays.

Overall, the findings of this study suggest that the concerned tourist is the most worried about safety aspects. Compared to the other groups, this segment comprises older tourists, which may explain their greater concern about hygiene measures. To target these tourists, organizations may focus on offering entire accommodation instead of shared apartments or rooms because these guests look for services like hotels. The indifferent tourists and the confident tourists, who make up the majority of the respondents, intend to travel to places close to home rather than in the surroundings, which is reinforced by Zenker and Kock (2020). These are tourists who choose sustainable solutions when travelling, which may be significant at the moment of their choice. As tourists are aware of sustainable business practices, they increasingly choose peer-to-peer accommodation in the sharing economy that presents sustainable practices. For instance, Fairbnb.coop, compared to Airbnb, represents a more benign business model, especially in terms of sustainability (Petruzzi et al., 2021). The forewarned tourists are also the tourists who worry about sustainability. However, they do not intend to have holidays close to home. Instead, they plan to travel abroad, which may be explained by their being the youngest profile. As frequent travellers, they require more information about hygiene measures, insurance against cancellation, and access to individual protective equipment.

These strategies are based on the design of safe experiences at peer-to-peer accommodation to give tourists the confidence to overnight there. Finally, this study represents an important undertaking from both the academic and organizational perspective in that it provides organizations with measures they can adopt in order to survive during and after the Covid-19 pandemic.

### Limitations and future research

This study uses data collected via a survey developed by a peer-to-peer accommodation organization. Thus, specifically with regard to the profile characterization, the data do not allow a deep analysis of country of destination and/or individual/family income. Another study limitation concerns the impossibility of controlling for potential variance due to the variety of data collection platforms. This study focuses on the perspective of tourists. Future research, therefore, should consider hosts’ opinions about safety measures and perhaps identify similarities and differences between the opinions of hosts and guests. Likewise, future research may also analyse guests’ opinions longitudinally throughout the evolution of the pandemic and/or based on the different practices already implemented. Furthermore, the views of governments on practices to be adopted by this type of organization may be worthwhile. As peer-to-peer accommodation in the sharing economy is still an emerging field of research, whose vulnerabilities were exposed during Covid-19, research is needed to support the future of the sector.
